# Risk Prediction Models for Mortality in Community-Acquired Pneumonia: A Systematic Review

**DOI:** 10.1155/2013/504136

**Published:** 2013-10-21

**Authors:** Chun Shing Kwok, Yoon K. Loke, Kenneth Woo, Phyo Kyaw Myint

**Affiliations:** ^1^Norfolk and Norwich University Hospital, Colney Lane, Norwich NR4 7UY, UK; ^2^Norwich Medical School, University of East Anglia, Norwich Research Park, Norwich NR4 7TJ, UK; ^3^School of Medicine & Dentistry, Division of Applied Health Sciences, University of Aberdeen, Aberdeen AB25 2ZD, UK

## Abstract

*Background*. Several models have been developed to predict the risk of mortality in community-acquired pneumonia (CAP). This study aims to systematically identify and evaluate the performance of published risk prediction models for CAP. *Methods*. We searched MEDLINE, EMBASE, and Cochrane library in November 2011 for initial derivation and validation studies for models which predict pneumonia mortality. We aimed to present the comparative usefulness of their mortality prediction. *Results*. We identified 20 different published risk prediction models for mortality in CAP. Four models relied on clinical variables that could be assessed in community settings, with the two validated models BTS1 and CRB-65 showing fairly similar balanced accuracy levels (0.77 and 0.72, resp.), while CRB-65 had AUROC of 0.78. Nine models required laboratory tests in addition to clinical variables, and the best performance levels amongst the validated models were those of CURB and CURB-65 (balanced accuracy 0.73 and 0.71, resp.), with CURB-65 having an AUROC of 0.79. The PSI (AUROC 0.82) was the only validated model with good discriminative ability among the four that relied on clinical, laboratorial, and radiological variables. *Conclusions*. There is no convincing evidence that other risk prediction models improve upon the well-established CURB-65 and PSI models.

## 1. Introduction

Community-acquired pneumonia (CAP) is common and associated with significant mortality [1–3]. Severity assessment is an important step in the management of CAP [4–6] because the early identification of individuals at high risk of death may help in deciding the site of care and the intensity of management [7]. Furthermore, subjective clinical judgment can underestimate pneumonia severity [8], and this may result in under-treatment and poor outcomes [9, 10]. Therefore, CAP risk prediction models have been developed to help clinicians predict pneumonia outcome and determine appropriate management more accurately.

The most widely known, well-validated, and commonly used risk prediction models are CURB-65 [3] and Pneumonia severity index (PSI) [11]. Recent systematic reviews have focused on assessing the comparative performance of these models [12, 13]. However, many other models have been developed, some of which are designed to predict mortality [14, 15], while others also include the need for ventilatory and vasopressor support [16–18]. The diverse and ever-increasing range of models may pose difficulties for clinicians who are attempting to choose a tool for use in their daily practice. To date, there has yet to be a clear consensus on the model that should be used [19], and no systematic attempt to compare the key characteristics and usefulness of the existing pneumonia scores has been made.

In this systematic review, we provide a comprehensive and up-to-date overview of the existing published risk prediction models for mortality in community-acquired pneumonia. We did not include scores which were designed to predict ventilatory and vasopressor support because of the inconsistency in decisions to provide these therapies depending on treatment site. We also aim to summarize the key features of each model such as variables used, risk stratification, and the comparative performance in terms of sensitivity, specificity, balanced accuracy, and area under the curve (AUC) values so that practitioners can make an informed choice.

## 2. Methods

### 2.1. Eligibility Criteria

We selected studies that were the first to report the derivation or validation of each risk prediction model for predicting mortality in CAP. There was no restriction on the type of study (prospective or retrospective) or country of origin. For pragmatic reason, we excluded studies that aimed to carry out further testing of risk models systems that had already been validated once and reported, as there are several validation studies for commonly used scores such as PSI and CURB-65. In such instances, we have used pooled data from published meta-analyses where available [12, 13]. Derivation studies were defined as studies which first reported the prognostic score. Validation studies were defined as studies which first tested the performance of a derived score in a separate cohort.

### 2.2. Search Strategy

We searched MEDLINE, EMBASE, and Cochrane Central Register of Controlled Trials with no date limitations in November 2011 using the search terms listed in Supplementary Material 1 available online at http://dx.doi.org/10.1155/2013/504136, without any language restriction. We also checked the bibliographies of included studies and recent review articles for relevant studies.

### 2.3. Study Selection and Data Extraction

Two reviewers (Chun Shing Kwok, Kenneth Woo) scanned all titles and abstracts to select studies that met the inclusion criteria. Full reports (where available) of potentially relevant studies were retrieved and independently checked by the other two reviewers (Yoon K. Loke, Phyo Kyaw Myint). Where there was any uncertainty or discrepancies, the article was discussed among the reviewers to determine if the studies should be included. We also contacted authors if there were any areas that required clarification. Data were collected using a standardized form by two authors independently (Chun Shing Kwok, Kenneth Woo), and this was checked by Yoon K. Loke. Data were collected on score name, setting for score application, year of study, country of origin, participant selection criteria, methodology for diagnosis of pneumonia, outcomes assessed, definition of severe pneumonia, participant characteristics, lost to followup in study, and the results. Data relating to study methodology were also collected for the quality assessment such as risk of confounding and statistical methods. The primary measure of interest was the area under the receiver operating curve (AUROC) as this reflects the overall discriminant ability of the risk prediction model; where this was not reported, we calculated balanced accuracy based on the following equation (sensitivity plus specificity) divided by two.

We also extracted results of existing meta-analyses on pneumonia risk prediction models [12, 13] to address the fact that both PSI and CURB-65 have been validated several times over, and we intended to present only the pooled data.

### 2.4. Assessment of Study Validity

Quality assessment was performed by Chun Shing Kwok using a methodological checklist for prognostic studies from the National Institute for Heath and Clinical Excellence [20]. Briefly, the checklist contains six components including study sample representative of population of interest, loss to followup unrelated to key characteristics, prognostic factor of interest, outcome of interest, potential confounders accounted for, and the appropriateness of statistical analysis.

### 2.5. Data Analysis

Due to the nature of this systematic review, we did not intend to conduct meta-analysis but planned to summarize the main findings descriptively in tables and figures. In particular, we evaluated key performance parameters (AUROC, balanced accuracy, sensitivity and specificity) for each scoring system and depicted this graphically according to the frequency of variables required for the calculation of the score. For these plots, we used validation study or meta-analysis results where available. We conducted additional subgroup analysis restricted to studies that used prospectively collected datasets, which may potentially be of greater validity than retrospective evaluations.

## 3. Results

**Figure 1:**
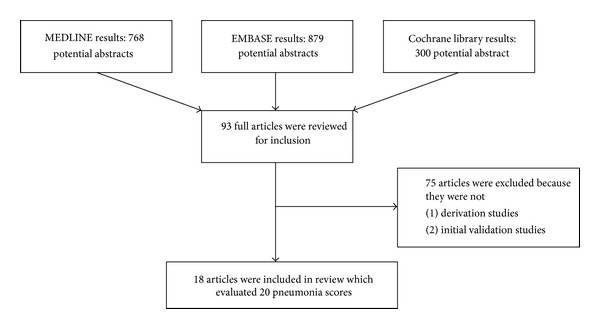
Search results and study selection.

From the 1,947 titles and abstracts, 93 articles were selected for detailed review (Figure 1). Of these, 20 different risk prediction models for mortality in pneumonia were described in 18 documents (including abstracts-only publications) between 1987 and 2011 (Figure 1) [6–8, 11, 14, 15, 21–32]. The list of excluded studies is shown in Supplementary Material 2. The detailed characteristics of studies and the description of individual models are shown in Table 1 and Supplementary Material 3, respectively. Aside from two [24, 28], all studies were conducted in emergency department settings. Diverse combinations of variables including patient characteristics, clinical features, laboratory results, radiological findings and physician judgments were considered across these models. Two studies used ICD-9 codes [11, 25] and one used ICD-10 codes to confirm pneumonia diagnosis [31]. One study [29] did not provide a formal definition as to how pneumonia was diagnosed.

### 3.1. Quality Assessment of Models

Study validity is summarized in Supplementary Material 4. One major limitation is that only 14 of the risk prediction models had validation data, whereas 6 reported findings from derivation studies (SOAR, AFSS, PARB, PIRO, CARSI, and CARASI) without further validation [24, 25, 28, 29, 32]. All studies had a study sample that appeared representative of the population of interest, with adequately defined outcomes. Mortality was the main outcome of interest in all but one study where a 30-day mortality and the need for oxygen therapy were combined [29]. The extent of lost to followup or missing data was unclear in the analysis for nine models (BTS 1, 2, 3, CURB, IDSA/ATS 2007, mATS, SOAR, A-DROP, and PARB) [6, 15, 22–24, 26, 29]. The impact of potential confounding factors was unclear in many studies, whereas eleven models (BTS 1, 2, 3, CURB, CURB-65, CRB-65, MRI, PSI, SOAR, AFSS, and PARB) [11, 14, 21–25, 29] used appropriate statistical methods (i.e., use of logistic regression models or statistical methods to choose factors that were most predictive of mortality) for the derivation of the prognostic score. Where statistical methods were not used to identify variables in the derivation of the models, some models were derived based on the hypothesis that certain variables may be correlated with death (e.g., shock index), while other models tested scores proposed from guidelines (e.g., ATS scores). One study was only available in the abstract form [29].

### 3.2. Variables Used in Risk Prediction Models

The frequency of variables which were used more than once in the models and their occurrence in individual scores is shown in Table 2. Variables were categorized into five groups: patient characteristics (age, gender, immunosuppression, and renal disease), clinical variables (pulse rate, blood pressure, respiratory rate, temperature, presence of shock, and confusion), laboratory measures (urea/blood urea nitrogen (BUN), white cell count, PaO_2_/SaO_2_, hematocrit, glucose, sodium, and pH), radiological findings (pleural effusion and multilobar pneumonia on chest X-ray), and physician judgment (need for mechanical ventilation). The four most commonly used variables (found in >10 scores) were confusion or altered mental status, respiratory rate, systolic blood pressure, and urea.

Some of the risk prediction models also required more complex concepts involving clinical interpretation and decision-making or even the results of other severity prediction tools. The MRI score included the Glasgow coma score, judgment on underlying ultimately or rapidly fatal illness, simplified acute physiology score, acute organ system failure, and ineffective initial antimicrobial treatment. The modified ATS score had major criteria of requirement for mechanical ventilation or septic shock, and the IDSA/ATS 2007 score included receipt of invasive mechanical ventilation and septic shock and the need for vasopressors. These models were therefore considered separately.

### 3.3. Risk Prediction Model Evaluation and Derivation and Validation Results

The results from the included derivation and validation studies are shown in Table 3. Supplementary Material 2 describes the individual severity scores according to the year of publication in chronological order.

### 3.4. Risk Prediction Models Using Only Clinical Variables

Four scores (BTS 1, CRB-65, CARSI, and CARASI) [21, 22, 32] were based on simple clinical measures that could be measured on first presentation in the community, with no requirement for laboratory or radiological testing. All were derived in the UK between 1987 and 2011. The number of variables ranged from three to six and respiratory rate was included in all scores. Of the two validated models, BTS1 and CRB-65 had fairly similar balanced accuracies (0.77 and 0.72 resp.), while CRB-65 was shown in the meta-analysis to have an AUROC of 0.78. Neither CARSI nor CARASI had been validated but the derivation studies had relatively low balanced accuracy (0.64) or AUROC (0.64) for both models. 

### 3.5. Risk Prediction Models Using Both Clinical Variables and Laboratory Testing

Nine prognostic models (BTS2, BTS3, CURB, CURB-65, A-DROP, CURB-age, SOAR, CURSI, CURASI) [21–24, 26, 31] were constructed using both clinical and laboratory parameters. They were developed in the UK between 1987 and 2010, except for A-DROP which was proposed by the Japanese Respiratory Society. All models were externally validated except for SOAR [24]. The number of variables ranged from three to six, and, respiratory rate was included in all models. Other commonly included variables were confusion and urea/blood urea nitrogen. CURB and CURB-65 had the best balanced accuracy (0.73 and 0.71, resp.). Here, AUROC was seldom reported amongst the modes but both CURB-65 (AUROC 0.79 from meta-analysis) and A-DROP (AUROC 0.85) showed reasonable discriminative ability. While A-DROP appears to have superior AUROC, we noted important quality issues regarding the absence of followup for vital status within the study (Supplementary Material 3) and lack of generalizability due to it being a retrospective, single-centre study of hospitalized patients.

### 3.6. Risk Prediction Models Using Clinical, Laboratorial, and Radiological Findings

Four models (PSI, AFSS, PIRO, and PARB) [11, 25, 28, 29] required radiological finding in their scoring system. These models were developed in the US, France, Spain, and Japan between 1996 and 2010; the number of variables ranged from four to twenty in these models [11]. The PSI is the only validated model here, with an AUROC of 0.82 in the meta-analysis. The performance of these models from derivation studies ranged from an AUROC of 0.75 for AFSS to 0.88 for the PIRO score.

### 3.7. Risk Prediction Models That Require Additional Clinical Decisions

Three models (MRI, mATS, and IDSA/ATS 2007) [6, 14, 15] gave weighting to clinical judgment, for example, that initial antimicrobial therapy was ineffective or that vasopressor therapy was needed for septic shock. These validated models were originated from the US and France and were principally designed for the prognostic use in intensive care settings or pneumonia cases that may need to be triaged to intensive care. The best performance here was achieved by the modified ATS score with a balanced accuracy of 0.94.

### 3.8. Summary of the Performance of Risk Prediction Models according to Number of Variables

**Figure 2:**
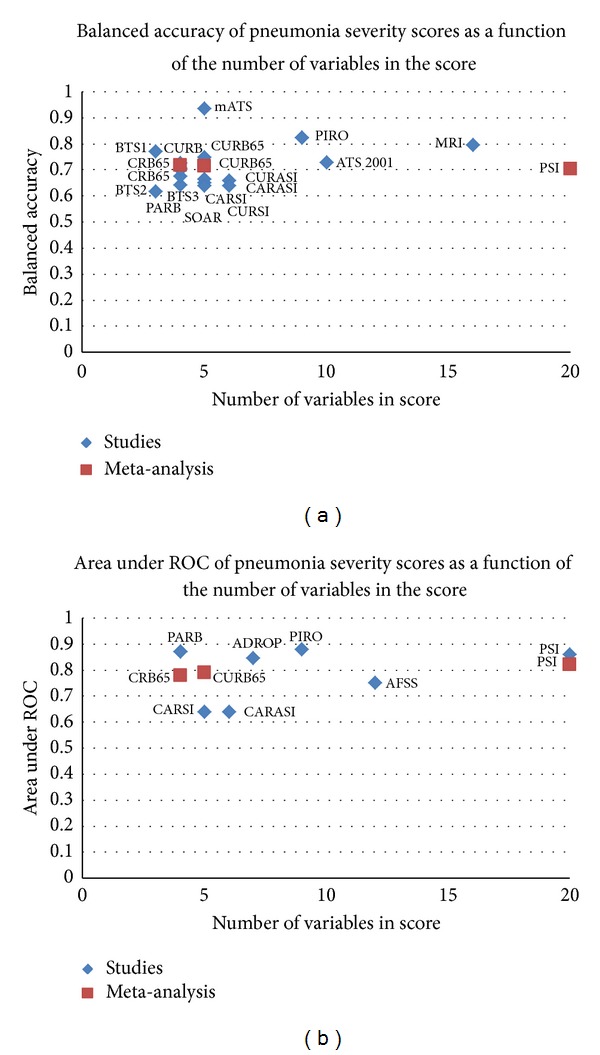
Balanced accuracy and area under ROC of pneumonia severity scores versus number of variables.

**Figure 3:**
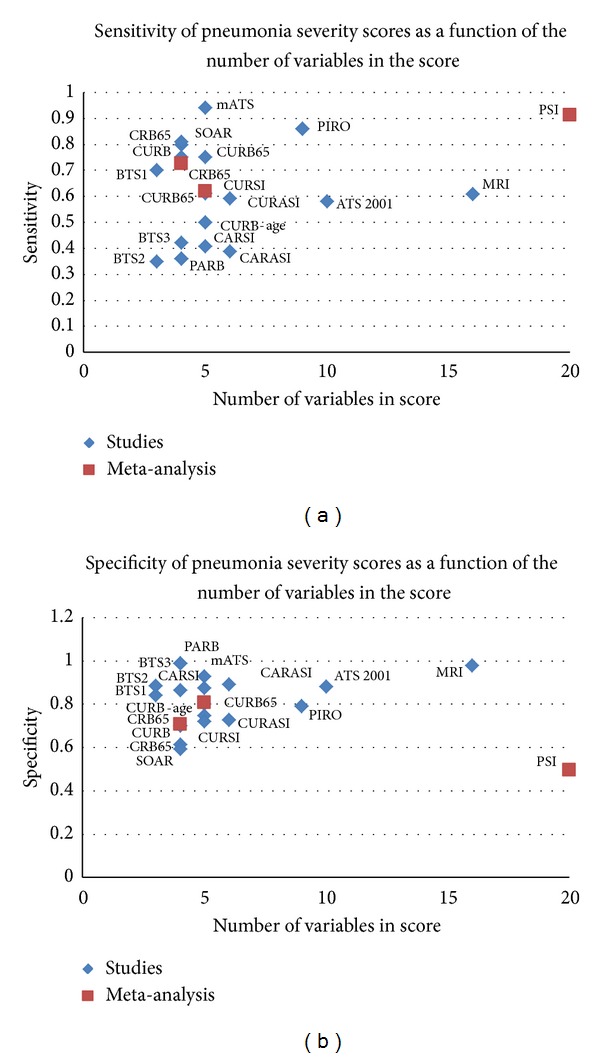
Sensitivity and specificity of pneumonia severity scores by a number of variables.

The comparative performance of the risk prediction models according to number of prognostic variables is summarized graphically in Figure 2 (balanced accuracy and AUC) and Figure 3 (sensitivity and specificity). Of the validated measures that are suitable for general clinical use, the CURB derivatives and PSI had the best balanced accuracies, and this is similarly reflected in the AUROC. Similarly, Figure 3 shows that PSI had amongst the highest sensitivity, but the tradeoff is apparent here in the lack of specificity for PSI as compared to other validated models such as CURB-65. We also conducted a subgroup analysis restricted to prospective studies as these may be of potentially higher validity than retrospective datasets (Supplementary Material 5).

## 4. Discussion

Our review systematically evaluates and summarizes 20 risk prediction models for mortality prediction which included variables required for score calculation in patients with pneumonia so that clinicians and policy makers (such as guideline committees and health services researchers) can make informed choices about the ease of use and comparative predictive ability. In these times of uncertainty in the health economy, the number and type of variables required for calculation need to be weighted up against the outright performance. Here, the ease of implementation, efficient resource utilization, and availability/simplicity of testing within healthcare setting (e.g., community centre, or emergency department, or intensive care unit) may represent influential factors in determining the suitability of a particular model.

We found that most of the published models (irrespective of complexity) yielded fairly similar performance with regard to balanced accuracy and AUC. While there may be some statistical differences in AUC, this may only have limited consequence when clinicians are making treatment decisions in individual patients. For instance, in Chalmer's meta-analysis, the respective AUCs indicate that the probability of PSI correctly discriminating between patients of differing severity was 0.82, whilst the corresponding figure for CURB-65 was 0.79. We have deliberately chosen to emphasize overall performance here with balanced accuracy or AUROC because while certain models may have demonstrably superior sensitivity, others had better specificity, thus illustrating the inevitable trade-off effect between sensitivity and specificity. The choice of appropriate model will therefore depend on whether healthcare teams place greater weight on sensitivity or specificity. Given the small differences between certain scoring systems, clinicians may equally prefer to either pragmatically adopt the simplest model (appropriate to their healthcare setting) or opt for the best established and widely validated systems. 

We presented both results for balanced accuracy and ROC in order to allow the comparison of the performance of each score. Balanced accuracy considers both the predictive value of sensitivity and specificity. While the ROC is a better measurement of predictive value than balanced accuracy, several studies reported sensitivity and specificity rather than ROC.

The majority of the studies were evaluated in hospital settings, but one study included both inpatients and outpatients and two studies were conducted in intensive care settings. The PSI was studied in both inpatient and outpatient settings which has an advantage because its findings can be generalisable to both of these settings [11]. Two studies, mortality risk index [14] and PIRO score [28], were conducted in intensive care settings. Community-based studies should be conducted in the future to include patients with less severe pneumonia. 

Our systematic review also identified some key gaps in the existing research. One particular issue is the lack of validation data for several models. Given the diversity of patient populations and the heterogeneity seen in the meta-analyses of PSI and CURB-65, there is no guarantee that a model that performs well in one setting will do equally well in a different setting. It would be very helpful if the profusion of recently proposed models (often based only on data from a single centre) could be compared directly against older versions in a large multicentre international cohort.

The existing studies do not report on acceptability, uptake, and clinical impact of risk prediction tool in the routine clinical management of patients with pneumonia. Perry et al. conducted a survey of emergency physicians' requirements for clinical decisions rule for acute respiratory illnesses [33], and they found that physicians wanted a highly sensitive rule with a median of 97.0% for respiratory conditions. The most sensitive tool here is PSI, which offers up to 90% sensitivity to help identify those at higher risk of death, but physicians in busy emergency departments may possibly find it too time-consuming and difficult to collect all of the variables (including detailed past medical history) for calculating the PSI. Hence, it appears from Perry's survey that there is a need for a score that is highly sensitive beyond what is currently available from any of the existing scoring systems. If the uptake and implementation of risk prediction tools in clinical decision are highly variable [34–37], then patients are unlikely to reap benefits from the current profusion of risk predictions tools. There is evidence to suggest that for the pneumonia severity index the uptake of this score and the scoring accuracy were low [38, 39]. Equally, it could be argued that the benefits of risk prediction models in reducing pneumonia morbidity and mortality need to be demonstrated in randomized controlled trials.

While the performance of a prediction rule is a major criterion for comparative superiority, simplicity is a very important determinant of potential clinical application. A survey conducted in Australia found that only 12% of respiratory physicians and 35% of emergency physicians reported using the PSI always or frequently even though it is recommended by the Australasian Therapeutic Guidelines [40]. Moreover, this study found that the majority of physicians were unable to accurately approximate the PSI scores and calculations of the simpler CURB-65 were more accurate [40]. This study concluded that it is recommended that a single, simple pneumonia severity score should be used in the assessment of CAP [40]. With the computer assisted programmes, PSI can be calculated easily and accurately. The pragmatic approach would be to use more complex scoring with high accuracy in resource-rich settings and to use alternative simpler scoring system in community or resource-poor settings. Our systematic review provides comprehensive comparison for clinicians to use any or a combination of scores of their choice in various health care settings.

Our review has a number of strengths. We conducted a systematic search to cover all scores including those that are established as well as those that have yet to be validated. Also, there was no restriction of the country of score origin and we were able to capture the scores from around the world. Our review also has a number of limitations, including difficulty in finding exact search terms to pick up this type of study. We only included initial derivation and first validation studies for the scores identified. Some of the scoring systems do not appear to have been validated yet. Here, there is a definite possibility of publication bias where studies showing the most favorable predictive ability were likely to be accepted for publication sooner than equivocal or less impressive data. In order to reduce the possibility of such bias, we were able to include two systematic reviews [12, 13] that examined the PSI and CURB scores (CRB-65, CURB, and CURB-65). 

Since there already exist established models (CRB-65, CURB-65, and PSI) with reasonable to good discriminative ability across a wide range of settings and only small incremental differences between these and newer scores, further research should mainly focus on why patients get misclassified and whether we can identify important variables within them to improve sensitivity of current models. Equally, the uptake of risk prediction models in routine clinical practice and any relationship with improved patient outcomes need to be rigorously assessed, perhaps through cluster-randomized controlled trials of different care pathways. These future trials should test if clinical decisions based on pneumonia scores are associated with better patient outcomes compared clinical decisions based on clinical judgment. Scores should also be tested in developing countries as pneumonia mortality is high in the regions. Eventually, the goal should be to clarify the entire pathway for community-acquired pneumonia management and the role of risk prediction models for each stage in the community, at the emergency department, on hospital wards, and in intensive care. 

## 5. Conclusions

Although there are a multitude of proposed risk prediction models, few have undergone proper validation, and no convincing evidence exists that the overall discriminative ability improves upon the well-established CURB-65 and PSI models. Future research should thus focus on randomized trials to test if clinical decision rules using existing risk prediction models and guided treatment pathways can significantly improve pneumonia outcomes.

## Supplementary Material

The supplementary material contains Appendix 1 (The search strategy), Appendix 2 (The list of excluded studies), Appendix 3 (The description of CAP scores), Appendix 4 (The quality assessment) and Appendix 5 (The sensitivity analysis of only prospective studies).

## Figures and Tables

**Table 1:** Characteristics of derivation and validation studies which predict pneumonia mortality.

Paper	Score	Design	Setting	Year	Country	Inclusion	CAP diagnosis	Mortality outcome
BTS 1987 [22] (derivation)	British Thoracic Society Score 1, 2, 3	Prospective	Hospital	November 1982 to December 1983	UK	Adults aged 15–74 years with pneumonia	Acute illness with radiological pulmonary shadowing which was neither preexisting nor of another known cause.	Mortality

Farr et al. 1991 [23] (validation)	British Thoracic Society Score 1, 2, 3	Retrospective	Hospital	January 1984 to 1986	United States	Adults aged from 15 to 80 years with the diagnosis of pneumonia	Acute respiratory illness contracted in the community and accompanied by a new radiographic infiltrate	Mortality

Leroy et al. 1996 [14]	Mortality risk index	Combined retrospective and prospective	ICU	Derivation January 1987–December 1992. Validation January 1993–December 1994	France	Adult patients aged >16 admitted to the intensive care and infectious disease unit with the diagnosis of CAP	Admission from home or a nursing home with the presence of pulmonary infiltrate on CXR and acute onset of clinical features of pneumonia	Mortality in ICU

Neill et al. 1996 [8]	CURB	Prospective	Hospital	July 1992 to 1993	New Zealand	Adults with pneumonia without severe immunosuppression	Acute illness radiographic pulmonary shadowing with neither preexisting nor another known cause	Mortality

Fine et al. 1997 [11]	Pneumonia severity index	Prospective	Hospital (inpatients and outpatients)	1989, 1991–1993	United States and Canada	Adults aged >18 years with diagnosis of pneumonia	ICD-9-CM diagnosis of pneumonia	30-day mortality

Lim et al. 2003 [21]	CURB-65, CRB-65	Retrospective analysis of prospectively collected data	Hospital	1998–2000	UK, New Zealand, and The Netherlands	Adults with CAP	Acute respiratory tract illness associated with radiographic shadowing on an admission chest radiograph	30-day mortality

Ewig et al. 2004 [15]	Modified American Thoracic Society Rule	Prospective	Hospital	June 1998–May 2001	Spain	All patients presenting with CAP in a university hospital between June 1998 and May 2001	New pulmonary infiltrate with symptoms and signs of a lower respiratory tract infection	30-day mortality

Myint et al. 2006 [24]	SOAR	Prospective	Hospital	NA	UK	Clinical features of pneumonia and new CXR shadow	Clinical features of pneumonia and new CXR shadow	42-day mortality

Myint et al. 2007 [27] (derivation)	CURB age	Prospective	Hospital	NA	UK	Clinical features of pneumonia and new CXR shadow	Clinical features of pneumonia and new CXR shadow	42-day mortality

Escobar et al. 2008 [25]	Abbreviated Fine Score	Retrospective	Hospital	2000–2002, 2004-2005	United States	All nonobstetric, nonpsychiatric patients aged >18 years with pneumonia	ICD codes defined by Fine et al	30-day mortality

Shindo et al. 2008 [26]	A-DROP	Retrospective	Hospital	November 2005–January 2007	Japan	Patients with CAP	Pneumonia in a patient who was not hospitalized and who was carrying on with activities of daily living	30-day mortality

Myint et al. 2009 [7] (validation)	CURB age	Prospective	Hospital	2006–2008	UK	Patients with CAP	Acute illness with clinical features of lower respiratory tract infection characterized by new radiographic shadowing	30-day mortality

Myint et al. 2009 [31] (derivation)	CURSI CURASI	Retrospective	Hospital	September 2004 to July 2005	UK	Patients with CAP	ICD-10 codes diagnosis of pneumonia	Inpatient mortality

Rello et al. 2009 [28]	PIRO score	Prospective	ICU	NA	Spain	Patients aged >18 years with pneumonia	Pneumonia confirmed by CXR and clinical findings	28-day mortality

Liapikou et al. 2009 [6]	IDSA/ATS 2007	Prospective	Hospital	January 2000–2007	Spain	Patients aged >15 years who were admitted to the emergency department for CAP in a university hospital from January 2000 through 2007	New pulmonary infiltrate on admission chest radiograph and symptoms and signs of lower respiratory tract infection	30-day mortality

Uchiyama et al. 2010 [29]	PARB	Retrospective	Hospital	March 2006 to November 2008	Japan	Adult patients with CAP	Unclear	30-day mortality or needing >2 weeks of oxygen therapy

Myint et al. 2010 [30] (validation)	CURSI, CURASI	Prospective	Hospital	2006–2008	UK	Clinical features of pneumonia and new CXR shadow	Clinical features of pneumonia and new CXR shadow	42-day mortality

Musonda et al. 2011 [32]	CARSI, CARASI	Prospective	Hospital	2008	UK	Patients with clinical and radiological features of CAP from 3 hospitals in the UK	Clinical features of pneumonia (cough, sputum, and shortness of breath, with or without fever) and new CXR shadow	30-day mortality

ICU: intensive care unit; CXR: chest X-ray; CAP: community-acquired pneumonia.

**Table 2:** Frequency of variables used in prognostic or severity scores in community-acquired pneumonia.

Score	Patient characteristics				Clinical variables						Laboratory measures							Radiological findings		Management
	Age	Gender	Immuno suppression	Renal disease	Pulse	BP	RR	Temp	Shock	Confusion	Urea/BUN	WCC	PaO_2_/SaO_2_	Haematocrit	Glucose	Sodium	pH	Pleural effusion	Multilobar pneumonia	Mechanical ventilation
BTS 1						+	+				+									
BTS 2						+	+			+										
BTS 3										+	+	+	+							
MRI			+						+			+							+	
CURB						+	+			+	+									
PSI	+	+		+	+	+	+	+		+	+		+	+	+	+	+	+		
CURB65	+					+	+			+	+									
CRB65	+					+	+			+										
mATS						+			+				+						+	+
SOAR	+					+	+						+							
AFSS					+	+	+	+		+	+		+	+	+	+	+	+		
A-DROP	+	+				+				+	+		+							
CURB-age	+					+	+			+	+									
PIRO score	+		+	+					+				+							
IDSA/ATS 2007						+	+	+	+	+	+	+	+						+	+
PARB							+				+							+		
CURSI					+	+	+			+	+									
CURASI					+	+	+	+		+	+									
CARSI	+				+	+	+			+										
CARASI	+				+	+	+	+		+										

BP: blood pressure; RR: respiratory rate; BUN: blood urea nitrogen; WCC: white cell count.

**Table 3:** Results of derivation and validation studies for pneumonia severity scores.

Paper	Score	Patients	Age	% male	Lost to followup	Results
BTS 1987 [22] (derivation)	British Thoracic Society Score 1, 2, 3	511 patients	48.4	60.5	28 lost to followup	Derivation: Score 1 (URB): 87.5% sensitivity, 78.7% specificity Score 2 (CRB): 39.1% sensitivity, 93.9% specificity Score 3 (COUW): 50% sensitivity, 89% specificity

Farr et al. 1991 [23] (validation)	British Thoracic Society Score 1, 2, 3	245 patients	58.9	55	None	Validation: Score 1 (URB): 70% sensitivity, 84.2% specificity, 28.6% PPV, 96.9% NPV, 82.3% overall accuracy Score 2 (CRB): 35% sensitivity, 88.5% specificity, 21.9% PPV, 93.7% NPV, 84% overall accuracy Score 3 (COUW): 42.1% sensitivity, 86.6% specificity, 24.2% PPV, 93.6% NPV, 82.4% overall accuracy

Leroy et al. 1996 [14]	Mortality risk index	460 patients, 335 derivation, 125 validation	62.5	64.3	None	Derivation: 62% sensitivity, 92% specificity, 74% PPV Validation: 61% sensitivity, 98% specificity, 92% PPV

Neill et al. 1996 [8]	CURB	255 patients	58	55	6 patients, no consent was obtained	Derivation: CURB: 95% sensitivity, 91% specificity, 22% PPV, 99% NPV BTS 1: 90% sensitivity, 76% specificity, 25% PPV, 99% NPV BTS 2: 65% sensitivity, 88% specificity, 33% PPV, 97% NPV BTS 3: 63% sensitivity, 88% specificity, 32% PPV, 97% NPV

Fine et al. 1997 [11]	Pneumonia severity index	14199 derivation, 38039 validation	NA	51	None	Derivation: PSI area ROC 0.84 Validation: PSI area ROC: MedisGroup cohort 0.83, PORT cohort 0.89

Lim et al. 2003 [21]	CURB-65, CRB-65	1068 patients	64	51.5	None	Derivation: CURB (>2): 75.4% sensitivity, 68.9% specificity, 20.5% PPV, 96.3% NPV CURB-65 (>3): 68.1% sensitivity, 74.9% specificity, 22.4% PPV, 95.7% NPV CRB-65 (>2): 76.8% sensitivity, 64.3% specificity, 18.6% PPV, 96.3% NPV Validation: CURB (>2): 75% sensitivity, 70.1% specificity, 20.5% PPV, 96.5% NPV CURB-65 (>3): 75% sensitivity, 74.7% specificity, 23.4% PPV, 96.7% NPV CRB-65 (>2): 80% sensitivity, 61.3% specificity, 17.6% PPV, 96.7% NPV

Ewig et al. 2004 [15]	Modified American Thoracic Society Rule	696 patients	67.8	66	21 patients had treatment setting not documented and were excluded	Validation mATS 94% sensitivity, 93% specificity, 49% PPV, 99.5% NPV, 93% overall accuracy BTS I 46% sensitivity, 87% specificity, 20% PPV, 96% NPV, 85% overall accuracy BTS II 53% sensitivity, 83% specificity, 19% PPV, 96% NPV, 81% overall accuracy mBTS 51% sensitivity, 80% specificity, 16% PPV, 96% NPV, 78% overall accuracy

Myint et al. 2006 [24]	SOAR	195 patients	77 (median)	57	None	Derivation: SOAR (≥2): 81.0% sensitivity, 59.3% specificity, 27.0% PPV, 94.4% NPV CURB (≥2): 81.5% sensitivity, 61.1% specificity, 25.9% PPV, 95.2% NPV CURB-65 (≥3): 81.5% sensitivity, 64.2% specificity, 27.5% PPV, 95.4% NPV CRB-65 (≥2): 85.2% sensitivity, 57.0% specificity, 24.5% PPV, 95.9% NPV

Myint et al. 2007 [27] (derivation)	CURB age	189 patients	75 (median)	56.1	None	Derivation: CURB age: 81.5% sensitivity, 74.1% specificity, 34.4% PPV, 96% NPV CURB-65: 81.5% sensitivity, 64.2% specificity, 27.5% PPV, 95.4% NPV

Escobar et al. 2008 [25]	Abbreviated Fine Score	11030 and 6147 patients	71.3	51.2	None	Derivation: AFFS: area ROC: inhospital mortality: 0.74 and 30-day mortality: 0.75

Shindo et al. 2008 [26]	A-DROP	371 patients	75	59.9	42 (lack data)	Validation: A-DROP: Area ROC 0.846 (0.790–0.903) CURB-65: Area ROC 0.835 (0.763–0.908)

Myint et al. 2009 [7, 31] (validation)	CURB-age	190 patients	76 (median)	53	None	Validation full cohort: CURB age: 50.0% sensitivity, 80.1% specificity, 50.0% PPV, 80.1% NPV CURB-65: 59.3% sensitivity, 75.7% specificity, 49.2% PPV, 82.4% NPV Validation for the elderly (>65 years): CURB age: 54.0% sensitivity, 70.6% specificity, 51.9% PPV, 72.3% NPV CURB-65: 64.0% sensitivity, 65.9% specificity, 52.5% PPV, 75.7% NPV

Myint et al. 2009 [7, 31] (derivation)	CURSI, CURASI	118	75 (median)	51.7	None	Only 1 patient died during hospital stay and the patient was scored severe by CURSI, CURASI, and CURB-65

Rello et al. 2009 [28]	PIRO score	529 patients	NA	NA	None	Derivation: PIRO: 86% sensitivity, 79% specificity, 61% PPV, 93% NPV, area ROC 0.88

Liapikou et al. 2009 [6]	IDSA/ATS 2007	2391 patients	66.7	61.4	289 missing data	Validation: ATS 2001: 58% sensitivity, 88% specificity

Uchiyama et al. 2010 [29]	PARB	243 patients	NA	NA	None	Derivation: PARB: 36% sensitivity, 99% specificity, area ROC 0.8705, accuracy 91.9%

Myint et al. 2010 [30] (validation)	CURSI, CURASI	190 patients	76 (median)	53	None	Validation full cohort: CURSI: 61.1% sensitivity, 72.1% specificity, 46.5% PPV, 82.4% NPV CURASI: 59.3% sensitivity, 72.8% specificity, 46.4% PPV, 81.8% NPV CURB-65: 59.3% sensitivity, 75.7% specificity, 49.2% PPV, 82.4% NPV Validation for the elderly (>65 years): CURSI: 62.0% sensitivity, 69.4% specificity, 54.4% PPV, 75.6% NPV CURASI: 60.0% sensitivity, 70.6% specificity, 54.5% PPV, 75.0% NPV CURB-65: 64.0% sensitivity, 65.9% specificity, 52.5% PPV, 75.7% NPV

Musonda et al. 2011 [32]	CARSI, CARASI	190 patients	76 (median)	53	None	Derivation: CARSI: 40.7% sensitivity, 87.5% specificity, 56.4% PPV, 78.8% NPV, 0.641 area ROC CARASI: 38.9% sensitivity, 89.0% specificity, 58.3% PPV, 78.6% NPV, 0.639 area ROC CURB-65: 59.3% sensitivity, 75.7% specificity, 49.2% PPV, 82.4% NPV, 0.675 area ROC

URB: urea, respiratory rate, blood pressure; CRB: confusion, respiratory rate, blood pressure; COUW: confusion, oxygen, urea, white cell count; PPV: positive predictive value; NPV: negative predictive value.
