# Supplementary material for: Risk Prediction Models for Mortality in Community-Acquired Pneumonia: A Systematic Review
Source: Biomed Res Int. 2013 Oct 21;2013:504136. doi: 10.1155/2013/504136 (PMC3817804; doi:10.1155/2013/504136)

**Appendix 1: Search strategy**

Database: Ovid MEDLINE(R) <1948 to November Week 3 2011>

Search Strategy:

--------------------------------------------------------------------------------

1 Pneumonia/ (33562)

2 pneumonia.mp. [mp=protocol supplementary concept, rare disease supplementary concept, title, original title, abstract, name of substance word, subject heading word, unique identifier] (96142)

3 (Risk or prognostic or prognosis or severity).mp. [mp=protocol supplementary concept, rare disease supplementary concept, title, original title, abstract, name of substance word, subject heading word, unique identifier] (1838408)

4 (Classification or score or index or assessment).mp. [mp=protocol supplementary concept, rare disease supplementary concept, title, original title, abstract, name of substance word, subject heading word, unique identifier] (1388864)

5 "Severity of Illness Index"/ (139262)

6 1 and 2 (33562)

7 4 and 5 (139262)

8 3 and 6 and 7 (768)

***************************

Database: Embase<1980 to 2011 Week 50>

Search Strategy:

--------------------------------------------------------------------------------

1 Pneumonia/ (79066)

2 pneumonia.mp. [mp=title, abstract, subject headings, heading word, drug trade name, original title, device manufacturer, drug manufacturer, device trade name, keyword] (152988)

3 (Risk or prognostic or prognosis or severity).mp. [mp=title, abstract, subject headings, heading word, drug trade name, original title, device manufacturer, drug manufacturer, device trade name, keyword] (2371470)

4 (Classification or score or index or assessment).mp. [mp=title, abstract, subject headings, heading word, drug trade name, original title, device manufacturer, drug manufacturer, device trade name, keyword] (2012491)

5 "Severity of Illness Index"/ (152352)

6 1 and 2 (79066)

7 4 and 5 (44835)

8 3 and 6 and 7 (879)

***************************

Cochrane library search and results

There are 300 results out of 661393 records for: "(Pneumonia) AND (Risk OR prognostic OR prognosis OR severity) AND (Classification OR score OR index OR assessment) in Title, Abstract or Keywords in Cochrane Central Register of Controlled Trials"

**Appendix 2: List of excluded studies**

| Score | Reason for exclusion | Reference |
| --- | --- | --- |
| SMART-COP | Not designed to predict mortality; designed to predict intensive respiratory or vasopressor support. | Charles PG, Wolfe R, Whitby M, et al (2008) SMART-COP: a tool for predicting the need for intensive respiratory or vasopressor support in community-acquired pneumonia. Clin Infect Dis 47(3):375-84. |
| CURX-80 | Not designed to predict mortality alone; designed to predict severe community acquired pneumonia. | Espana PP, Capelastegui A, Gorordo I, et al (2006) Development and validation of a clinical prediction rule for severe community-acquired pneumonia. Am J RespirCrit Care Med 174:1249-1256. |
| CORB | Not designed to predict mortality alone; designed to predict death, ventilator or inotropic support. | Buising KL, Thursky KA, Black JF, et al (2007) Identifying severe community-acquired pneumonia in the emergency department: a simple clinical prediction tool. Emerg Med Australas 19:418-26. |
| I-ROAD | Not designed for community-acquired pneumonia alone; also includes healthcare-associated pneumonia. | Matsunuma R, Ohkuni Y, Nakashima K, et al (2010) I-Road could be efficient in predicting severity of community acquired pneumonia (CAP) or healthcare-associated pneumonia (HCAP). ERS Annual Congress, Barecelona. |

**Appendix 3: Prognostic or severity scores in community acquired pneumonia**

| **Score** | **Score full name** | **Score criteria** | **Range** | **Severe definition** | **Reference** |
| --- | --- | --- | --- | --- | --- |
| BTS 1, BTS 2, BTS 3 | British Thoracic Society Score 1, British Thoracic Society Score 2, British Thoracic Society Score 3. | BTS score 1: RR ≥30 breaths/min; diastolic BP ≤60 mmHg; blood urea >7 mmol/l.  BTS score 2: RR ≥30 breaths/min; diastolic BP ≤60 mmHg; confusion.  BTS score 3: confusion; PaO2 ≤6.6 kPa; blood urea >7 mmol/L; white cell count ≤10x10^9^/L or lymphocyte ≤1x10^9^/L. | Severe and non-severe. | Severe for BTS 1 and BTS 2 is 2 out of 3. Severe for BTS 3 is 3 out of 4. | BTS 1987 |
| MRI | Mortality Risk Index | 16 predictors: Aspiration pneumonia (-0.37); grading of sepsis >11 (-0.2); antimicrobial combination (-0.01); Glasgow score >12+mechanical ventilation (MV) (+0.09); serum creatinine>15 mg/1 (+0.22); chest involvement shown by X-ray >3 lobes (+0.28); shock (+0.29); bacteraemia (+0.29); initial MV (+0.29); underlying ultimately or rapidly fatal illness (+0.31); Simplified Acute Physiology Score _>12 (+0.49); neutrophil count <3500/mm^3^ (+0.52); acute organ system failure score _>2 (+0.64); delayed MV (+0.67); immunosuppression (+1.38); and ineffective initial antimicrobial therapy (+1.5). | Score -0.5 to 6.0 | Severe defined as MRI ≥2.5. | Leroy 1996 |
| CURB | - | CURB: confusion; urea >7 mmol/L; RR ≥30 breaths/min; diastolic BP ≤60 mmHg. | Severe and non-severe. | Severe for CURB ≥2 points. | Neill 1996 |
| PSI | Pneumonia Severity Index | Step 1: age >50 years, each of five coexisting illnesses (neoplastic disease, congestive heart failure, cerebrovascular disease, renal disease, and liver disease), and each of five physical examination findings (altered mental status; pulse (≥125 per minute); RR (≥30 breaths/min); systolic BP (<90 mm Hg); and temperature,<35°C or ≥40°C)  Step 2: in addition to the 11 factors identified in step 1, 2 demographic factors (male sex and nursing home residence) and 7 laboratory or radiographic findings (BUN, ≥30 mg/dl [11 mmol/l]; glucose concentration, ≥250 mg/dl [14 mmol/l]; haematocrit, <30 percent; sodium concentration, <130 mmol/l; PaO_2_, <60 mm Hg; arterial pH, <7.35; and pleural effusion). | Class I-V based on points | Severe defined as class IV and V. | Fine 1997 |
| mATS rule | Modified American Thoracic Society rule | At least two of three minor criteria assessed at admission (systolic BP <90 mm Hg; multilobar (>2 lobes) involvement; PaO_2_/FiO_2_<250); or one of two major criteria assessed at admission or during follow up (requirement for mechanical ventilation or septic shock). | Severe and non-severe. | Severe: 2 of 3 Minor criteria or or 1 of 2 Major criteria. | Ewig 2003 |
| CURB-65,  CRB-65 | - | CURB65: Confusion; Urea >7 mmol/l; RR >30 breaths/min; and low BP (diastolic BP <60 mm Hg or systolic BP <90 mm Hg)); age ≥65  CRB65: Confusion; RR >30 breaths/min; and low BP (diastolic BP <60 mm Hg or systolic BP <90 mm Hg)); age ≥65; | Severe and non-severe. | Severe for CURB-65 ≥3 points and for CRB-65 ≥2 points. | Lim 2003 |
| SOAR | - | Systolic BP <90 mmHg, oxygenation (PaO2:FiO2 <250), age (≥65 years), RR (≥30/min). | 0 to 4 | Severe defined as ≥2. | Myint 2006 |
| AFSS | Abbreviated Fine Severity Score | 12 predictors: Altered mental status; RR >30 breaths/min; Systolic BP <90 mm Hg; Temperature <35°C; Pulse >125 beats/min; Arterial pH <7.35; BUN >30 mg/dL; Sodium <130 mEq/L; Glucose >250 mg/dL; Haematocrit <30%; PaO_2_<60 mm Hg; Pleural effusion. | 0 to 180 | Unclear | Escobar 2008 |
| A-DROP | - | Age (male≥70 years, female≥75 years); Dehydration (BUN ≥ 210 mg/L); Respiratory failure (SaO_2_ ≤ 90% or PaO_2_≤ 60 mm Hg); Orientation disturbance (confusion); and low BP (systolic BP ≤90 mmHg) | 0 to 5 | Severe defined as ≥3. | Shindo 2008 |
| CURB-age | CURB-age | Confusion (1 point); urea >7 mmol/l but ≤11 mmol/l or >11 mmol/l (1 point); RR ≥30 breaths/min (1 point); either diastolic BP ≤60 mm Hg or systolic BP ≤90 mm Hg (1 point); age >65 and ≤85 (1 point), or >85 (2 points) | 0 to 7 | Severe defined as >4 points. | Myint 2007  Myint 2009 |
| PIRO score | - | Predisposition: Comorbidities (chronic obstructive pulmonary disease or immunocompromise); age >70 yrs  Insult: Bacteraemia; Multilobar opacities in chest radiograph  Response: Shock; Severe hypoxemia  Organ dysfunction: Acute renal failure; acute respiratory distress syndrome | 0 to 8 | Severe defined as 4 or more points. | Rello 2009 |
| IDSA/ATS 2007 | IDSA/ATS 2007 | Minor: Respiratory rate ≥30 breaths/min; PaO2/FiO2 ≤250; Multilobar infiltrates; Confusion and/or disorientation; Uraemia (BUN level ≥20 mg/dL); Leukopenia (WBC count <4 x 10^9^ cells/L); Thrombocytopenia (platelet count <100 x 10^9^ platelets/L);  Hypothermia (core temperature <36°C); Hypotension (systolic BP <90 mm Hg; requiring aggressive fluid resuscitation);  Major: Receipt of invasive mechanical ventilation, Septic shock with the need for vasopressors | Severe and non-severe. | Severe: 1 of 2 Major criteria or 3 of 9 Minor criteria. | Liapikou 2009 |
| PARB score | - | One point for each of the following: Pleural effusion, albumin <3.0 g/dl, RR >30/min, BUN >25 mg/dL. | 0 to 4 | Unclear | Uchiyama 2010 |
| CURSI, CURASI | - | CURSI: confusion, urea >19.6 mg/dL (7 mmol/L), RR rate ≥30 breaths/min and SI value of >1.0 (1 point each) (maximum possible score=4)  CURASI: shock index replaced with an adjusted shock index and using the same scoring system. Shock index: pulse rate divided by the systolic BP. Adjusted shock index: account for heart rate rise associated with the rise in body temperature. To adjust for this physiological phenomenon we deduct 10 points of the heart rate for every 1.0 °C increase in the patient's temperature above 37.0 °C before calculating the SI | 0 to 4 | Severe defined as ≥2. | Myint 2009  Myint 2010 |
| CARSI, CARASI | - | CARSI: confusion (1 point), age <85 but ≥65 (1 point), age ≥85 (2 points), RR ≥30/min (1 point), and an shock index value of >1.0 (1 point) (maximum possible score=5)  CARASI: shock index was replaced with an adjusted shock index and using the same scoring system. | 0 to 5 | Severe defined as score ≥3. | Musonda 2011 |

RR = respiratory rate, PaO2 = arterial oxygen tension, FiO2 = fraction of inspired oxygen, GCS = Glasgow coma score, SAPS = Simplified Acute Physiology Score, OFS = Organ System Failure, BP = blood pressure, BUN = blood urea nitrogen, COPD = chronic obstructive pulmonary disease, CXR = Chest X-ray

**Appendix 4: Quality assessment of pneumonia severity scores**

| **Score** | **Study sample represents population of interest** | **Loss to follow up unrelated to key characteristics** | **Prognostic factor of interest** | **Outcome of interest** | **Potential confounders accounted for** | **Statistical analysis is appropriate** |
| --- | --- | --- | --- | --- | --- | --- |
| BTS 1, BTS 2, BTS 3 | Yes: Adults 15-74 years. | Unclear: 28 lost to follow up. | Yes: Score with clinical and laboratory variables. | Yes: mortality. | Unclear: Many factors considered but not compared for severe and non-severe group. | Yes: Stepwise logistric regression in derivation. |
| MRI | Yes: Adult patients admitted to intensive care with diagnosis of CAP. | Yes: None reported. | Yes: Score with clinical, laboratory and radiological variables. | Yes: mortality. | No: Multiple factors associated with mortality considered but not included in score. | Yes: Cannonical discriminant analysis in derivation. |
| CURB | Yes: Adults with pneumonia. | Unclear: 6 patients no consent was obtained. | Yes: Score with clinical and laboratory variables. | Yes: mortality. | Unclear: Unclear if other variables were different between the severe and non-severe group. | Yes: Stepwise logistic regression in derivation. |
| PSI | Yes: Adult inpatients with CAP. | Yes: None reported. | Yes: Score with several stages combining clinical, laboratory and radiological variables. | Yes: 30-day mortality. | Yes: Derivation involved evaluating predictive value of many candidate predictors. | Yes: Logistic regression in derivation. |
| IDSA/ATS 2007 | Yes: Adults admitted with diagnosis of CAP. | No: 289 patients had missing data. | Yes: Scores with clinical, laboratory and radiological variables. | Yes: mortality. | No: Baseline characteristic differences for severe and non-severe group. | No: Based on guidelines. |
| mATS rule | Yes: Patients admitted with CAP. | Unclear: 21 patients had treatment setting not documented and were excluded. | Yes: Scores with clinical, laboratory and radiological variables. | Yes: mortality. | Unclear: Unclear of other variables were different between the severe and non-severe group. | No: Modification of score based on guidelines. |
| CURB-65,  CRB-65 | Yes: Adults admitted to hospital with CAP. | Yes: None reported. | Yes: Score with clinical and laboratory variables. | Yes: 30-day mortality. | Yes: Derivation included 12 potential predictors. | Yes: Backward logistic regression in derivation. |
| SOAR | Yes: Pneumonia among elderly with age ≥65 years. | Unclear: 6 patients included in analysis did not have complete CURB-65 criteria. | Yes: Score with clinical and laboratory variables. | Yes: 6 week mortality. | Unclear: Unclear of other variables were different between the severe and non-severe group. | Yes: Backward stepwise logistic regression in derivation. |
| AFSS | Yes: Non-obstetric and nonpsychiatric adults hospitalized with pneumonia. | Yes: None reported. | Yes: 12 variables out of the 19 in the pneumonia severity index. | Yes: Inhospital mortality, 30-day mortality. | Unclear: Unclear if other variables were different between the severe and non-severe group. | Yes: Logistic regression analysis. |
| A-DROP | Yes: Patients with CAP admitted to hospital. | No: 42 patients missing. Also, patients who had no missing records indicating death were analysed as survived. | Yes: Score with clinical and laboratory variables. | Yes: 30-day mortality. | Unclear: Unclear of other variables were different between the severe and non-severe group. | No: Based on guidelines. |
| CURB-age | Yes: Patients with CAP. | Yes: None reported. | Yes: Score with clinical and laboratory variables. | Yes: Mortality at 42 days. | No: Multiple baseline variables were significantly different between severe and non-severe pneumonia. | No: Modification of score based on hypothesis. |
| PIRO score | Yes: Adults with pneumonia admitted to intensive care unit. | Yes: None reported. | Yes: Score with clinical, laboratory and radiological variables. | Yes: Death at 28 days. | Unclear: Baseline characteristics considered elsewhere. | Unclear: Score derivation methods unclear. |
| PARB score | Yes: Adult inpatients with CAP. | Unclear: Not reported. | Yes: Score with clinical, laboratory and radiological variables. | Unclear: Combined outcome of 30-day mortality and needing more than 2 weeks of oxygen therapy. | Unclear: Baseline characteristics were not described. | Yes: Logistic regression analysis for significant predictors. |
| CURSI, CURASI | Yes: Adults patient with CAP. | Yes: Not reported. | Yes: Score with clinical and laboratory variables. | Yes: 6-week mortality. | No: Baseline difference between severe and non-severe group. | No: Hypothesis driven. |

**Appendix 5: Sensitivity analysis restricted to prospective studies**


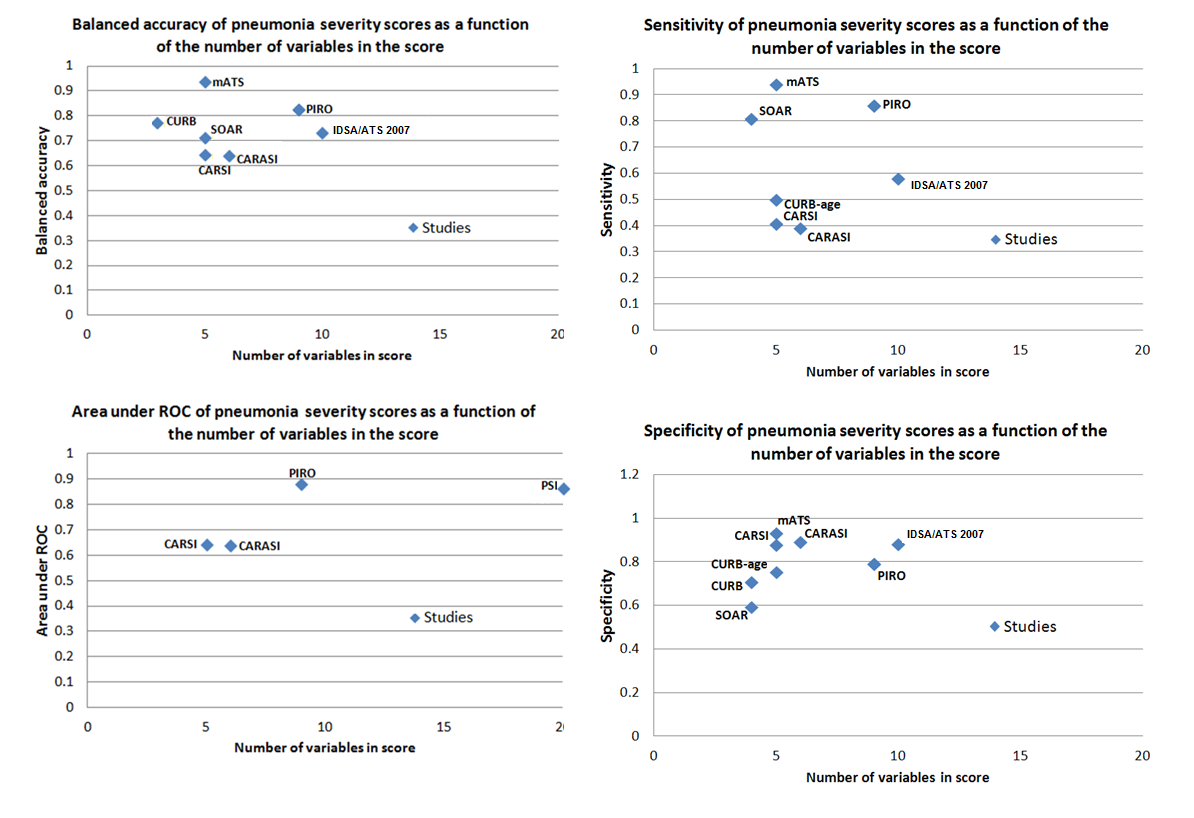

Supplement: Supplementary file 1 — The supplementary material contains Appendix 1 (The search strategy), Appendix 2 (The list of excluded studies), Appendix 3 (The description of CAP scores), Appendix 4 (The quality assessment) and Appendix 5 (The sensitivity analysis of only prospective studies). [file 504136.f1.docx]
